# Designing, Validation, and Feasibility of Integrated Approach of Heartfulness Meditation and Yoga Protocol (IAHFNM & YP) for Hypertensive Participants

**DOI:** 10.1155/2024/9289232

**Published:** 2024-07-04

**Authors:** Pooja S. Singh, Veronique Nicolai, Yogesh Patil, Neelam Yeram, Bhusan Bhukte, Kapil Thakur, Mitesh Thakker, Haresh Mehta, Mansee K. Thakur

**Affiliations:** ^1^ Department of Medical Biotechnology Central Research Laboratory MGM School of Biomedical Sciences MGM Institute of Health Sciences, Navi Mumbai, India; ^2^ Department of International Yoga Academy Heartfulness Institute Kanha Shanti Vanam, Hyderabad, Telangana, India; ^3^ Department of Biochemistry MGM School of Biomedical Sciences Kamothe, Navi Mumbai, Maharashtra, India; ^4^ Heartfulness Meditation Centre SRCM Heartfulness Institute, New Panvel, India; ^5^ Department of Medicine MGM Medical College & Hospital MGMIHS, Navi Mumbai, Maharashtra, India; ^6^ Department of Non-Invasive Cardiology Raheja Super Speciality Hospital, Mumbai, Maharashtra, India

**Keywords:** feasibility, heartfulness meditation, hypertension, pilot study, validation, yoga

## Abstract

The module was designed and developed at Heartfulness Institute, Kanha Shanti Vanam, Hyderabad. The Department of Medicine, MGM Medical College & Hospital, MGMIHS, Navi Mumbai, carried out the validation and subsequently pilot-tested on volunteers. Forty experts were selected to validate the contents of IAHFNM & YP which was designed after a thorough review of meditation and yoga literature. A total of 23 items were included, and each item was rated as essential or not essential by the experts, based on which the content validity ratio (CVR), Item-Level Content Validity Index (I-CVI), and Scale-Level Content Validity Index Average (S-CVI/Ave) were calculated. Reliability analysis and a pilot study for the feasibility of IAHFNM & YP for hypertensive patients were also done. All 23 practices exhibited significant CVR (≥ 0.29), I-CVI (> 0.79), and S-CVI/Ave (> 0.9); thus, the tool was found to have valid contents. Cronbach's alpha value for the tool was 0.95 which was highly reliable. Feasibility analysis in hypertensive participants showed that the tool is reliable and implementable. The IAHFNM & YP tool designed for hypertensive patients is valid, reliable, and feasible. The patients showed a willingness to continue with heartfulness meditation and yoga practices for participation in research for a longer duration. Further studies to confirm the tool's efficacy should be conducted with a large sample size.

**Trial Registration:** CTRI/2024/01/061035

## 1. Introduction

Globally and in India, the prevalence of noncommunicable diseases (NCDs) is rising. They are major obstacles to sustainable development and the reduction of poverty [1]. One risk factor for cardiovascular morbidity and mortality is hypertension. According to disability-adjusted life years, it is projected to have contributed 7% of the disease burden and 9.4 million fatalities in 2010 [2]. Current modern lifestyle habits and dietary patterns lead to several consequences in which body homeostasis is challenged. Alterations in homeostasis result in increased blood pressure (BP) and associated disorders such as hypertension and cardiovascular diseases [3–5].

Among various modifiable direct and indirect risk factors, a sedentary lifestyle resulting from emerging globalization and modernization has been identified as one of the primary causes of chronic diseases [5]. Evidence has also shown the association of psychological factors, such as negative affect, suppressed hostility, and emotional defensiveness, with elevated BP and increased risk of hypertension [6, 7]. The American College of Cardiology, American Heart Association, and European Society of Hypertension guidelines recommend lifestyle interventions for the prevention and treatment of hypertension [8, 9]. At present, hypertension management strategies are aimed at reducing BP and including lifestyle modifications such as dietary patterns, physical activity, and stress reduction, along with pharmacological treatment [3, 7].

Nonpharmacological interventions play a significant role in the prevention and management of hypertension, as demonstrated by a plethora of research studies on lifestyle interventions, including single-component interventions such as exercise, diet, breathing exercises, and yoga, as well as multicomponent lifestyle interventions. These lifestyle modifications have been shown to effectively control BP and reduce cardiovascular morbidity and mortality [7, 10, 11].

Currently, there is growing evidence to support the use of several complementary and alternative medicine activities to control high BP, including yoga, certain relaxation techniques, and meditation. Yoga and meditation are ancient techniques used as nonpharmacological mind-body interventions and have been studied for their potential benefits in individuals with hypertension and their effectiveness in improving cardiovascular functions [10–14]. Deep breathing exercises, including yogic techniques, have been found to reduce stress and manage hypertension [12, 14]. These techniques have been used for thousands of years as practical, safe, and effective tools for stress reduction and health management strategies [12, 14].

One such meditation technique is heartfulness meditation, which is rooted in the Raja yoga tradition and referenced from the Yoga Sutras [15–17]. This technique is based on centering focus on the heart as a source of inner guidance and spiritual growth. The core of heartfulness practice is the uniqueness of yogic transmission, which has benefits such as stress reduction, improved emotional well-being, increased self-awareness, and enhanced spiritual development. This technique of meditation is aimed at attaining a balanced state of mind that eventually improves physical health, psychological health, burnout, sleep quality, and stress [18, 19]. The beneficial effects of heartfulness meditation may also contribute to improving health status in individuals suffering from hypertension and related diseases [13, 14].

Today, there is a need for developing and testing the feasibility, acceptability, and efficacy of an integrated approach that involves interventions involving both yoga and meditation practices, which would aid in the prevention and control of hypertension by controlling considerable risk factors and prove vital in combatting the high-risk burden of hypertension globally [4, 20]. To date, very few studies have focused on the development of feasible and effective yoga and meditation protocols for hypertension treatment. However, the development and efficacy of an Integrated Approach of Heartfulness Meditation and Yoga Protocol (IAHFNM & YP) for hypertension treatment are unresolved. Combined therapy involving yoga and heartfulness meditation techniques, depicting the transition between asanas and meditation, would enable a simultaneous upswing of physical, mental, and spiritual well-being. In this study, we aimed to design and validate a feasible, cost-effective tool for meditation and yoga intervention (i.e., an IAHFNM & YP) for efficiently improving the lifestyles of individuals with mild and moderate hypertension.

### 1.1. Study Objective

The primary aim of this study was to design, validate, and conduct a feasibility analysis of the IAHFNM & YP for hypertensive patients.

## 2. Materials and Methods

**Figure 1:**
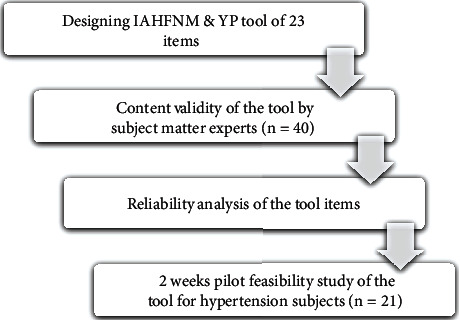
Flowchart of steps in the design, validation, and feasibility analysis of IAHFNM & YP for hypertensive participants.

A one-arm feasibility study was conducted to assess the research protocol and provide proof of concept for the proposed integrated approach. The research adhered to CONSORT (Consolidated Standards of Reporting Trials) reporting guidelines. The study was approved by the institutional ethics committee of the MGM Institute of Health Sciences, Navi Mumbai (MGMIHS/R&D/ECRHS/03/2023/185), and CTRI approval was obtained on 1 January 2024. The study was conducted in strict adherence to the principles of the World Medical Association Declaration of Helsinki [21]. Signed informed permission was acquired by each subject. The design, validation, and feasibility of the IAHFNM & YP for hypertension (Figure 1) were carried out via the following steps.

### 2.1. Phase I: Development of IAHFNM & YP

This study began with a thorough systematic literature review of traditional and contemporary texts on yoga and meditation, which was conducted under the guidance of experienced yoga and meditation trainers, respectively. Yoga practices have been taken from major yoga literature, such as the Hatha Yoga Pradipika, Gheranda Samhita, and Patanjali Yoga Sutras [15, 22–24]. Integrated Approach to Yoga Therapy for Positive Health, Pranayama - The Arts and Science, Yoga Darsana, Sūryanamaskara (sun salutation), and yoga therapy series-yogic management of ailments are some of the sources from which the yoga protocol has been compiled [25–33]. A variety of search engines, including PubMed, Google Scholar, Web of Science, Medline, and Scopus, were used to find research articles on the use of alternative remedies for hypertension as well as current scientific reviews of the condition [29–33]. The search terms “Meditation”, “Yoga”, “Hypertension”, “Stress”, “Pranayama”, and “Hathayoga” were utilized to determine the beneficial effects of yoga therapy on hypertension and stress. These items were carefully chosen considering the foundational principles and distinctive features to develop the IAHFNM & YP for hypertension, which comprises 23 practices encompassing mind-body loosening, asanas, pranayama, relaxation, meditation, and rejuvenation.

### 2.2. Phase II: Content Validation of the IAHFNM & YP by Subject Matter Experts (SMEs)

The developed IAHFNM & YP was shared with 40 SMEs as a Google Form with a brief explanation of the study objectives for rating the practices included in the scale. The experts were asked to rate the 23 practices in the proposed IAHFNM & YP tool on a two-point scale—1 indicating *yes (essential)* and 2 indicating *no (not essential)*.

1. *Yes (essential)*: important in improving any symptoms or the quality of life of patients with hypertension.
2. *No (not essential)*: not useful in improving any symptoms of general well-being or hypertension.

The extent to which items in the questionnaire/instrument correspond can be measured using content validity [34, 35]. This can be evaluated using content validity ratio (CVR), Item-Level Content Validity Index (I-CVI), and Scale-Level-CVI (S-CVI). The I-CVI is calculated by dividing the total number of experts who rate an item as “very relevant/essential” by the total number of experts. The S-CVI is obtained by dividing the total number of items by the sum of the I-CVIs [36, 37].

The CVR for the practices included in the tool was calculated using Lawshe's formula, and a cutoff value of 0.29 was obtained by referring to Lawshe's table according to the number of experts included in the study [35].

(1) $$\begin{array}{c} \text{CVR}=\frac{\left(\text{Ne}-N/2\right)}{N/2} \end{array}$$

where Ne is the number of SMEs indicating *yes (essential)* and *N* = 40 is the total number of SMEs.

Furthermore, the I-CVI was calculated for individual items in the scale, the acceptance value of which is > 0.79 for individual items.

(2) $$\begin{array}{c} \text{I}‐\text{CVI}=\frac{\text{Ne}}{N}. \end{array}$$

The validity of the tool was confirmed by calculating the Scale-Level Content Validity Index Average (S-CVI/Ave) by using the following formula:

(3) $$\begin{array}{c} \text{S}‐\text{CVI}/\text{Ave}=\frac{\sum \text{I}‐\text{CVI}}{I} \end{array}$$

where *I* = Total number of item in scale. Total number of item in the study (*I*) = 23.

A value of S‐CVI/Ave ≥ 0.9 indicates excellent content validity [35].

Practical and qualitative inputs for validation of the module were also obtained from the experts, such as the duration, number of rounds of each practice, similarity in certain practices, order, and modifications within the practice, to accommodate the criteria.

### 2.3. Phase III: Feasibility of IAHFNM & YP for Hypertension Treatment

Feasibility was assessed on the basis of the attrition rate, retention rate, and subjective difficulty during the practice. Research participants were also requested to opine on an overall 15-day IAHFNM & YP intervention.

#### 2.3.1. Inclusion and Exclusion Criteria

Individuals with a diagnosis of hypertension, aged 21 to 60, were included in the feasibility study with their informed consent. Participants with a history of severe disability, those who had undergone recent spinal or abdominal surgery, those who had previously practiced yoga, those undergoing physiotherapy, those experiencing heart failure or vertigo, pregnant women, and those with any physical impairment or medical condition that could potentially interfere with the administration of tools were excluded from the study.

#### 2.3.2. Participants

A convenience sampling method was used to recruit participants from Heartfulness Centre in New Panvel, India, in our study. Heartfulness Centre sent out invitations to participants. A note was put up on the notice board, and everyone who was a member of the Heartfulness Centre and had high BP received a WhatsApp text message encouraging them to volunteer for the study. Participants received comprehensive information on the study, including its goals, methods, any drawbacks, and advantages. Participants were required to answer questions regarding their age, gender, BP, lifestyle choices, comorbidities, and socioeconomic status. Of the approximately 34 hypertensive people recruited in the study, 13 were nonadherent and 21 adherents (six dropped out and seven gave incomplete data). Pilot research was conducted on these 21 people who had been clinically diagnosed with hypertension in order to assess the acceptability and viability of the validated IAHFNM & YP tool. The mean age of the patients was 35.5 ± 10.7 years.

#### 2.3.3. Intervention

Hypertensive participants underwent intervention via the IAHFNM & YP protocol for 2 weeks at the Heartfulness Centre, Panvel, Navi Mumbai; there were three yoga sessions under supervision each week. Participants attended the IAHFNM & YP sessions in comfortable clothing with a minimum fasting period of approximately 1 h before the intervention. To follow meditation practices, the participants were initially introduced to In-Person Orientation Training for Heartfulness Meditation by a heartfulness certified trainer, which involved three consecutive days for 2 h per day along with yoga asanas.

1. Day 1: yoga asanas, pranayama, and heartfulness meditation for morning practice.
2. Day 2: review day 1, morning practice and training in the evening practice of rejuvenation.
3. Day 3: review morning and evening practice and follow-up: recordings and access to the live sessions.

#### 2.3.4. Morning Practice (45 min)

The 45-min sessions involved in the morning practice were structured into six subsections: (1) centering with a yogic prayer and yogic breathing for 5 min, (2) three standing warm-up exercises for 5 min, (3) 13 yoga asanas for 20 min, and (4) Shavasana with a guided heartfulness relaxation technique for 5 min. (5) After relaxation, participants were asked to sit in a comfortable posture such as Sukhasana or Vajrasana to practice 10 rounds of Anuloma-Viloma and five rounds of Shitali pranayama, 5 min each to prepare their mind and body to enter the meditative state. (6) The final step of the morning practice was dhyana (5 min of heartfulness meditation) which was guided by a certified heartfulness trainer to a participant three times per week. Participants practiced guided meditation in the online mode on other days during follow-up.

#### 2.3.5. Evening Practice (15 min)

Evening practice is divided into two subscales consisting of 5 min of Bhramari pranayama, which relaxes the mind and body and promotes positive thinking, followed by 10 min of rejuvenation (i.e., heartfulness cleaning) which is one of the characteristic practices of heartfulness tradition.

All the participants were assessed for their ability to perform all the practices under the supervision of heartfulness certified meditation and yoga instructor for specified durations. Lastly, 21 patients completed the intervention and post assessment. Six patients dropped out during the intervention phase due to time constraints. None of the dropouts were attributed to adverse effects of the intervention.

### 2.4. Statistical Analysis

Descriptive analysis was performed on the sociodemographic data of the SMEs and hypertensive participants. The content validity of the IAHFNM & YP tool was calculated using formulae in Microsoft Excel. The reliability of the tool was determined by IBM-SPSS software (version 26.0).

## 3. Results and Discussion

### 3.1. Design and Validation of the IAHFNM & YP

The IAHFNM&YP consisted of loosening and breathing practices, yoga postures, *pranayama*, relaxation methods, and heartfulness meditation. Forty experts (47.5% male and 52.5% female) who validated the tool ranged in age from 35 to 72 years (mean 50.68 years, standard deviation (SD) ± 11.13). These experts were from various prestigious institutes across India, such as SVYASA University of Yoga, Kaivalyadhama Institute of Yoga, All India Institute of Medical Sciences (AIIMS) Gujarat, and AIIMS Hyderabad. They are skilled in a number of areas, including yoga, ayurveda, physiology, cardiology, mindfulness, and heartfulness training. Approximately 42.5% of the experts had 7–15 years of experience, 47.5% of the experts had 16–30 years of experience, and 10% of the experts had > 30 years of experience.  Table 1 indicates the CVR value for each IAHFNM & YP item and time allotted for each item. The results show that all items with CVRs ≥ 0.29 are valid and essential for the tool, as Lawshe's CVR critical value for 40 SMEs is ≥ 0.29. Thus, all 23 practices were retained in the designed IAHFNM & YP tool. The I-CVI and S-CVI/Ave values were found to be above the acceptable values (i.e., > 0.79 and > 0.9, respectively), for all the practices, showing the excellent content validity of the items included in the tool.

### 3.2. Reliability Analysis of the IAHFNM & YP

The internal consistency of the 23 items incorporated in the IAHFNM & YP tool was assessed by reliability analysis. In Table 2, Cronbach's alpha value obtained for the IAHFNM & YP tool is > 0.70, which indicates that the items in the scale have high internal consistency and that the intervention tool is reliable.  Table 3 indicates that the deletion of any item from the tool cannot further improve Cronbach's alpha value for the scale; hence, each item is essential and reliable.

The baseline sociodemographic characteristics of the 21 participants who were included in the study are shown in Table 4. Some participants had a history of comorbidities such as diabetes (38.1%), dyslipidemia (23.8%), anxiety (33.3%), and depression (23.8%). All participants were nonsmokers, and 19 participants were nonalcoholic.

The acceptability and feasibility of the IAHFNM & YP tool were assessed using a two-point Likert self-efficacy questionnaire, which assessed the feasibility of each item for participants as “yes” or “no.” The data obtained from participants showed that all the practices included in the tool were feasible, and participants were willing to continue their involvement in the study for a longer duration to inculcate these practices in their daily lifestyle. Participants did not experience any adverse side effects when performing the tool practices.

The acceptability of each practice included in the IAHFNM & YP tool is shown in Table 5, according to which almost all the practices in the tool were feasible for more than 50% of the participants except for some asanas, such as Ardha Ustrasana, Shashankasana, and Uttanapadasana.

## 4. Discussion

Together with medication, lifestyle modifications are necessary for the proper management of hypertension and BP control. A mere 2- or 3-mmHg reduction in the average BP of the population could dramatically reduce the occurrence of cardiovascular disorders [38]. Numerous studies have demonstrated that in addition to pharmaceutical medications, lifestyle changes, complementary therapies, and other interventions are necessary for the management of hypertension. When these elements are implemented early in people with hypertension and prehypertension, future complications including cardiovascular problems may be avoided [39, 40]. Particularly noted were integrated yoga and meditation practices that lower BP [32].

IAHFNM & YP was created after a careful analysis of the literature on classical yoga. Asanas (physical postures), pranayama (breathing exercises), and dhyana (meditative meditation) were all incorporated within the module. Experts in yoga validated the module. Lawshe's CVR formula was the analytical technique employed. In the final module, only practices that were deemed essential were included. The experts also determined that the entire module was essential. The experts recommended that the yoga practices included in the developed and validated IAHFNM & YP tool are easy to practice and must be customized based on individual preferences. They also suggested the addition of relaxation sessions between yoga practices for better performance. A few SMEs recommended that the duration of these practices be adjusted according to the feasibility of the participants; for example, one could start with a shorter duration initially and then gradually extend it based on the subject's stamina, ultimately reaching an optimal duration under individualized supervision for better effects.

This study's module validation approach was in line with other studies on the creation and validation of yoga modules. A yoga module for heart disease was created and validated by Sha et al. [41]. A yoga module on Parkinson's illness was validated by a study conducted by Kakde et al. [42]. Patil et al. yoga module for persistent lower back pain was created and shown effective [43].

The methodology used in the pilot phase of the study was consistent with earlier researches that tested feasibility of customized yoga modules [44]. The pilot reliability of the IAHFNM & YP tool was analyzed based on the responses obtained from the hypertensive participants toward the developed integrated scale. In Tables 2 and 3, we observed that all the items incorporated in the tool had high internal consistency, indicating the high reliability of the tool for further validation and feasibility studies.

The responses from the participants in the feasibility screening showed that the scale is acceptable and feasible for hypertensive patients of all age groups. Almost all the participants were able to practice scale items as per allotted duration i.e., 60 min (45 min of morning practice, 15 min of evening practice) each day. But less than half of the participants said that Ardha Ustrasana, Shashankasana, and Uttanapadasana were difficult. This study shows that yoga can effectively lower BP in people with hypertension. There was also no evidence of stress or full-day alertness. Similar results were reported by additional trials [45, 46].

In a similar vein, another research found that yoga successfully lowers BP in both hypertensive and normotensive individuals. But it is challenging to suggest a particular style of yoga due to the diversity of yoga practices and the paucity of research on the practice's long-term effects [47]. Another review offers favorable but equivocal results about the efficacy of yoga in treating hypertension [48].

The present study is unique since it has developed a validated IAHFNM & YP module [49]. The study's strengths are as follows: (i) the IAHFNM & YP was carefully developed based on both ancient and modern yoga literature; (ii) it was an integrated module incorporating physical postures, breathing exercises, and meditation techniques; (iii) a sufficient number of SMEs validated the chosen practices, and the responses were examined using a reliable statistical tool; and (iv) critically, the validated module was put through a pilot study with a paired sample pre-post measurement design. The module proved effective in lowering BP, affecting physical fitness, activity levels, and self-esteem, according to the pilot study. The study's shortcomings and weaknesses were as follows: (i) the sample size of the pilot study was small (*n* = 21), (ii) the period of intervention was 2 weeks which is short, and (iii) no follow-up study was conducted to ascertain long-term benefits. Future studies could be undertaken using a randomized control trial design with larger sample size and a longer period of intervention. This study could also be explored in national and international population. It is significant to remember that none of the pilot research participants reported any severe side effects on their own. These results provide evidence in favor of evaluating this module in prospective studies including hypertension patients.

The findings from this study may be useful for medical health professionals to manage hypertension effectively in an orderly and systematic manner. Further studies to assess the efficacy of the tool through randomized trials would be beneficial in clinical research and may become a therapeutic option for hypertension.

## 5. Conclusions

Based on established research, an IAHFNM & YP demonstrated the advantages and demonstrated that it was a strong and advantageous intervention for hypertension. All of the experts that validated the protocol thought that most of these activities were appropriate. The pilot study indicated that participants reported no unanticipated bad events and considered it useful. It was suggested that there should be more training sessions, and the pilot study suggested that there might not be enough practice sessions. The study demonstrates the face validity and content validity of the IAHFNM & YP program. Long-term influence requires longer training. Clinical validation is necessary for future research.

## Figures and Tables

**Table 1:** Content validation of IAHFNM & YP tool by SMEs.

**IAHFNM & YP item**				**Activity practice**		**Activity duration**		**Decision**
**Practice activity**			**Duration (min)**	**CVR**	**I-CVI**	**CVR**	**I-CVI**	
A	Morning yoga and meditation	Centering + yogic prayer + yogic breathing	05	1.00	1.00	0.95	0.98	Retained

B	Standing warm-up	Tadasana	05	0.90	0.95	0.50	0.75	Retained
		Triyak Tadasana		0.90	0.95	0.50	0.75	Retained
		Katichakrasana		0.90	0.95	0.50	0.75	Retained

C	Yogasana	Padahastasana half	20	0.95	0.98	0.95	0.98	Retained
		Marjariasana		0.95	0.98	0.95	0.98	Retained
		Ardha Ustrasana		0.95	0.98	0.95	0.98	Retained
		Shashankasana		0.95	0.98	0.95	0.98	Retained
		Baddha Konasana		0.95	0.98	0.95	0.98	Retained
		Gomukhasana		0.90	0.95	0.50	0.75	Retained
		Vakrasana		0.95	0.98	0.95	0.98	Retained
		Bhujangasana		0.95	0.98	0.95	0.98	Retained
		Shalabhasana		0.95	0.98	0.95	0.98	Retained
		Uttanapadasana		0.95	0.98	0.95	0.98	Retained
		Setu Bandhasana		0.95	0.98	0.95	0.98	Retained
		Pawanamuktasana		0.95	0.98	0.95	0.98	Retained
		Jathara Parivartanasana		0.95	0.98	0.95	0.98	Retained

D	Relaxation	Heartfulness relaxation in Savasana	05	1.00	1.00	0.70	0.85	Retained

E	Pranayama	Anuloma-Viloma pranayama	05	0.90	0.95	0.85	0.93	Retained
		Shitali pranayama		0.90	0.95	0.85	0.93	Retained

F	Heartfulness meditation	Heartfulness meditation	05	0.70	0.85	0.50	0.75	Retained

G	Evening practice	Bhramari pranayama	05	1.00	1.00	0.95	0.98	Retained
		Heartfulness cleaning	10	0.80	0.90	0.65	0.83	Retained

S-CVI				—	0.93	—	0.91	—

**Table 2:** Reliability analysis of IAHFNM & YP tool by hypertensive participants' response.

**Statistics**	**Value**
No. of items	23
Scale mean (SD)	30.57 (7.06)
Scale variance	49.86
Scale Cronbach's alpha	0.95^∗^

^∗^Cronbach's alpha value > 0.70 is considered a good scale.

**Table 3:** Reliability analysis of IAHFNM & YP tool for each item by hypertensive participant's response.

**Feasibility and acceptability study of the validated IAHFNM & YP tool**				
**IAHFNM & YP practice**		**Scale mean if item deleted**	**Scale variance if item deleted**	**Cronbach's alpha if item deleted**
Morning yoga and meditation	Morning yoga and meditation	29.33	46.23	0.94

Standing warm-up	Tadasana	29.29	44.71	0.94
	Triyak Tadasana	29.24	47.59	0.95
	Katichakrasana	29.24	44.69	0.94

Yogasana	Padahastasana half	29.14	45.83	0.94
	Marjariasana	29.14	44.73	0.94
	Ardha Ustrasana	28.86	46.93	0.95
	Shashankasana	29.00	45.50	0.94
	Baddha Konasana	29.29	45.01	0.94
	Gomukhasana	29.19	46.36	0.95
	Vakrasana	29.29	44.61	0.94
	Bhujangasana	29.38	44.95	0.94
	Shalabhasana	29.38	44.95	0.94
	Uttanapadasana	28.90	47.39	0.95
	Setu Bandhasana	29.24	45.69	0.94
	Pawanamuktasana	29.24	45.69	0.94
	Jathara Parivartanasana	29.14	45.13	0.94

Relaxation	Savasana	29.33	45.23	0.94

Pranayama	Anuloma-Viloma pranayama	29.38	44.95	0.94
	Shitali pranayama	29.38	44.95	0.94

Heartfulness meditation	Heartfulness meditation	29.43	47.86	0.95

Evening practice	Bhramari pranayama	29.33	44.93	0.94
	Heartfulness cleaning	29.43	47.86	0.95

**Table 4:** Demographic data of hypertensive participants for feasibility study.

**Characteristics**	**Category**	**Value**
Age (years)	Mean (SD)	55.24 (16.19)
	Range	21–76

Gender: *n* (%)	Female	10 (47.6)
	Male	11 (52.4)

Sedentary lifestyle: *n* (%)	Mild	6 (28.6)
	Moderate	13 (61.9)
	Strenuous	2 (9.5)

Blood pressure: Mean (SD)	Systolic BP (mmHg)	152 (17.19)
	Diastolic BP (mmHg)	102.57 (13.71)

**Table 5:** Frequency of acceptance of the IAHFNM & YP items by hypertensive participants.

**IAHFNM & YP item**		**No. of participants voted “yes”**	**Acceptance (%)**
Morning yoga and meditation	Morning yoga and meditation	16	76.2

Standing warm-up	Tadasana	15	71.4
	Triyak Tadasana	14	66.7
	Katichakrasana	14	66.7

Yogasana	Padahastasana half	12	57.1
	Marjariasana	12	57.1
	Ardha Ustrasana	06	28.6^∗^
	Shashankasana	09	42.9^∗^
	Baddha Konasana	15	71.4
	Gomukhasana	13	61.9
	Vakrasana	15	71.4
	Bhujangasana	17	81.0
	Shalabhasana	17	81.0
	Uttanapadasana	07	33.3^∗^
	Setu Bandhasana	14	66.7
	Pawanamuktasana	14	66.7
	Jathara Parivartanasana	12	57.1

Relaxation	Savasana	16	76.2

Pranayama	Anuloma-Viloma pranayama	17	81.0
	Shitali pranayama	17	81.0

Heartfulness meditation	Heartfulness meditation	18	85.7

Evening practice	Bhramari pranayama	16	76.2
	Heartfulness cleaning	18	85.7

^∗^Acceptance was less than 50% in the study population.

## Data Availability

This published paper include all the data which was collected during research work. All data will be made available upon request to the corresponding author.
